# Individualized rs-fMRI reveals brain-circuit heterogeneity and predicts early recurrence in trigeminal neuralgia

**DOI:** 10.3389/fpsyt.2026.1778601

**Published:** 2026-02-20

**Authors:** Zhongshuai Ma, Zhengming Wang, Xu Su, Min Cheng, Zhijia Wang, Chao Du, Yu Tian

**Affiliations:** 1 Department of Neurosurgery, China-Japan Union Hospital of Jilin University, Changchun, Jilin, China; 2 Department of Trauma Center, China-Japan Union Hospital of Jilin University, Changchun, Jilin, China; 3 Department of Neurosurgery, Xinqiao Hospital, Army Medical University, Chongqing, China; 4 Department of Radiology, China-Japan Union Hospital of Jilin University, Changchun, Jilin, China

**Keywords:** individualized analysis, neuromodulation, precision biomarker, recurrence, regional homogeneity, resting-state fMRI, risk stratification, trigeminal neuralgia

## Abstract

**Objective:**

To identify abnormal brain regions in patients with trigeminal neuralgia (TN) and screen for specific regions that can predict short-term recurrence after percutaneous radiofrequency ablation (RFT).

**Methods:**

Resting-state functional magnetic resonance imaging (rs-fMRI) was used to identify differential brain regions in TN patients. An individualized rs-fMRI approach was applied to screen for recurrence-related brain regions in patients undergoing RFT. Among these, regions with a 100% recurrence rate were classified as high-risk recurrence regions. Treatment outcomes and changes in these differential brain regions were observed postoperatively.

**Results:**

Thirty TN patients exhibited 19 differential brain regions. Four of these—Rolandic_Oper_L, Cerebellum_9_L, Lingual_R, and Calcarine_L—were newly identified as abnormal regions in TN. Among the 15 patients who underwent RFT, 15 potential recurrence-related regions were found. Six of these—contralateral Insula_L, Fusiform_L, Vermis_3, and Temporal_Sup_L; ipsilateral Cerebellum_3_R; and ipsilateral Fusiform_R (when involving V1 division pain)—were identified as high-risk recurrence regions. Follow-up scans confirmed that these recurrence-related differential brain regions were either eliminated or attenuated after surgery.

**Conclusion:**

Patients with trigeminal neuralgia exhibit abnormalities in multiple brain regions. These findings demonstrate that individualized functional imaging biomarkers provide an effective framework for stratifying the risk of early postoperative recurrence. Specifically, abnormalities in the Insula_L, Fusiform_L, Cerebellum_3_R, Temporal_Sup_L, Vermis_3, and Fusiform_R can be defined as high-risk brain regions for predicting short-term recurrence after radiofrequency ablation.

## Introduction

Trigeminal neuralgia (TN) is a severe neurological disorder characterized by paroxysmal pain (1, 2). The primary surgical treatment modalities for TN include: microvascular decompression(MVD), percutaneous radiofrequency thermocoagulation (RFT), and gamma knife radiosurgery (GKS) (1, 3, 4). The coexistence of multiple surgical approaches for TN stems from the distinct advantages and disadvantages associated with each method. RFT offers a higher safety profile, with no reported mortality or severe disability, making it suitable for patients without confirmed vascular compression on CNV, elderly patients, or those with comorbidities precluding craniotomy. Nevertheless, the relatively high postoperative recurrence rate significantly impacts the efficacy of RFT (5–9).

There are some reports about magnetic resonance imaging (MRI) and postoperative recurrence rate of RFT. 5%-10% of patients with severe vascular compression (Grade III) fund by MRI experience recurrence within 6 months postoperatively (1, 10–12). Forevermore, our previous work revealed that when classifying TN patients using MR Diffusion Tensor Imaging (MR-DTI) findings, the probability of recurrence within 6 months after RFT was 10% for the L-FA type but as high as 60% for the N-FA type (13, 14). While these studies have identified some factors contributing to short-term recurrence after RFT, the complete set of risk factors predicting short-term recurrence for individual TN patients remains incompletely defined. Predicting and preventing short-term recurrence is therefore a critical concern.

Functional magnetic resonance imaging (fMRI) is another MR technique and can analyze changes in brain activity induced by pain stimuli (15).Abnormal functional brain regions in TN are critical findings on fMRI. Key questions warranting attention are whether the abnormal activation in specific brain regions can serve as novel predictive indicators for short-term recurrence following RFT (16–18).

In this study, while applying traditional fMRI methodology, we also first established a novel rs-fMRI approach: individualized comparison of each TN patient against a normal control group. This new individualized method – individualized rs-fMRI (irs-fMRI) – holds promise for providing clinically meaningful predictive indicators of surgical treatment outcomes for individual TN patients.

Using conventional rs-fMRI methods, this study identified four novel abnormal brain regions. Furthermore, applying the irs-fMRI method to 15 consecutive patients undergoing percutaneous stereotactic radiofrequency ablation(PSR)with DTI guiding surgery, we discovered that abnormal activation in six brain regions, including Insula_L, Fusiform_L, Vermis_3, Cerebellum_3_R, Temporal_Sup_L, and Fusiform_R, can serve as predictive indicators for short-term recurrence after RFT in TN patients.

Beyond group-level effects, TN likely reflects meaningful inter-individual heterogeneity in pain-related brain circuits. Precision neuropsychiatry emphasizes linking biological heterogeneity to individualized biomarkers that can support patient stratification and targeted clinical decision-making. In chronic pain conditions such as TN, brain networks involved in sensory processing, salience, affect, and memory may jointly shape symptom severity and treatment response. Therefore, an individualized rs-fMRI strategy that evaluates each patient against a normative reference could provide a practical imaging biomarker for identifying patients at higher risk of early relapse and for informing precision-oriented follow-up and targeted intervention planning.

## Methods

### Study subjects

Subjects: A total of 30 patients with TN (12 males, 18 females; age 62.93 ± 11.52 years) were enrolled from China-Japan Union Hospital of Jilin University. The inclusion criteria were as follows: (1) Diagnosis of TN meeting the criteria of the International Classification of Headache Disorders, 3rd edition (ICHD-3) (19); (2) Seeking treatment at the Department of Neurosurgery, China-Japan Union Hospital of Jilin University (Changchun City, Jilin Province) between March 2021 and December 2024.The inclusion criteria for the healthy control group were: (1) No history of neurological or psychiatric disorders; (2) No history of trigeminal nerve-related pain. Exclusion criteria for both groups: (1) Contraindications for MRI/fMRI scanning; (2) History of other facial pain syndromes or prior invasive surgical interventions for trigeminal neuralgia.

**Figure 1:**
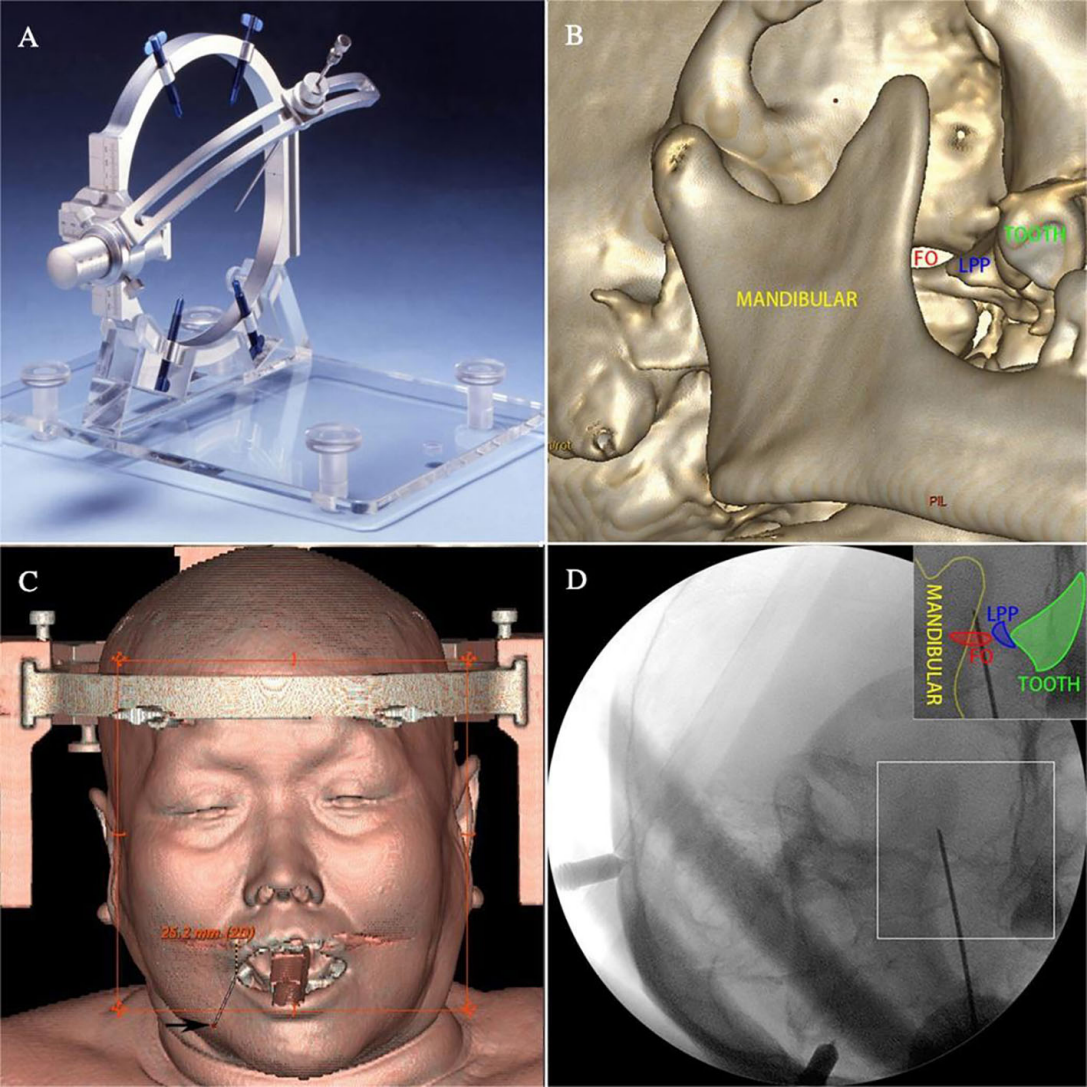
PSR surgical equipment and procedure. **(A)** Komai’s frame-based CT-Stereotactic system. **(B)** 3D CT reconstruction image for FOT localization. **(C)** Application of the air-to-air meeting technique to complete the puncture. **(D)** X-ray C-arm verification of the puncture needle reaching the FOT.

All participants provided written informed consent. Written consent for publication of a potentially identifiable facial photograph (**Figure 1**) was obtained from the patient.

Grouping: All 30 TN patients (VAS score 4-10) constituted the TN observation group (TN group). Fifteen patients who underwent PSR-DTI surgery constituted the PSR observation group (PSR group). Within the PSR group, patients experiencing postoperative recurrence formed the recurrence subgroup, while patients achieving complete pain relief formed the non-recurrence subgroup. Thirty healthy individuals served as the control group.

**Figure 2:**
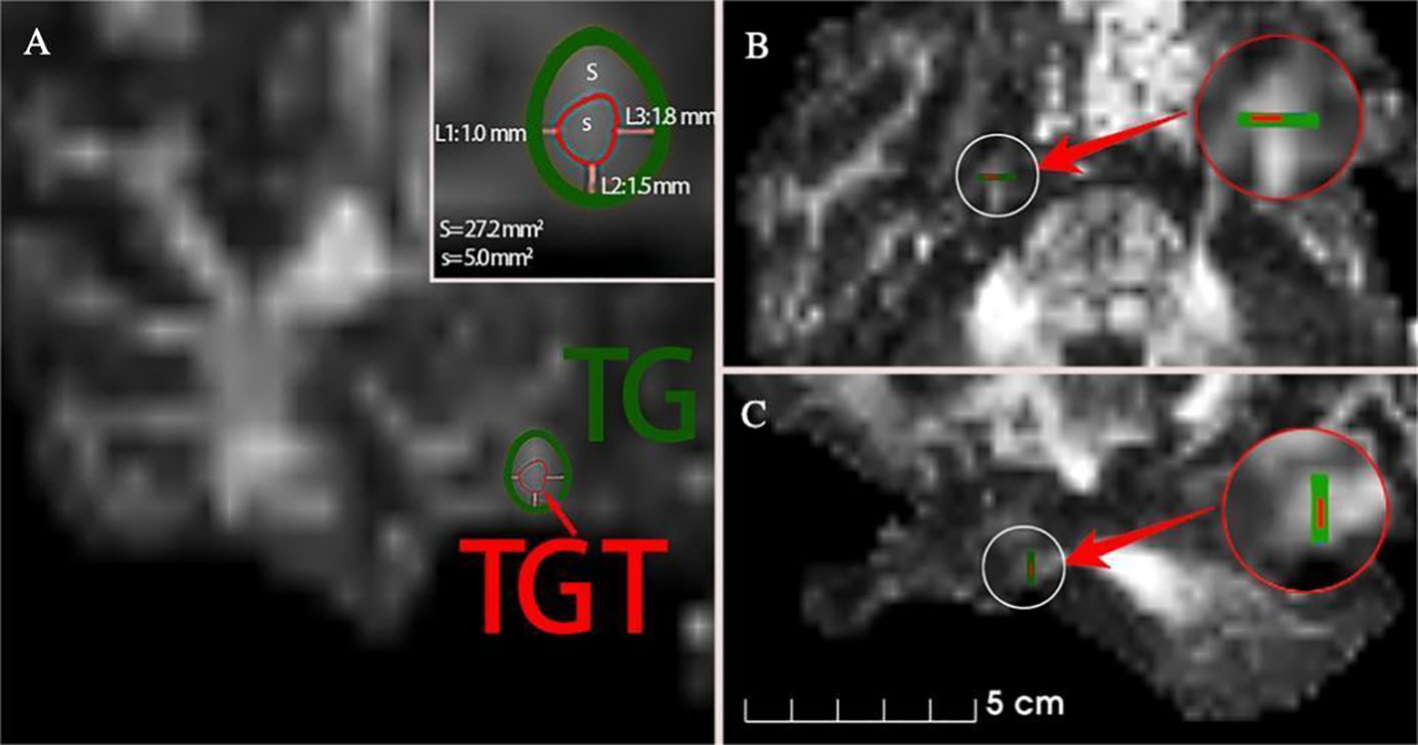
MR-DTI Imaging for Trigeminal Ganglion Treatment Target (TGT) (**A, B**) Axial and coronal images showing the TGT located medially within the center of the Trigeminal Ganglion (TG). **(C)** Coronal image showing the TGT located inferiorly within the TG.

PSR Surgical Method: PSR surgery was performed using the Komai’s frame-based CT-Stereotactic system (Mizuho Medical Innovation, Tokyo, Japan) (**Figure 1**) and guided by MR-DTI images for aim at the trigeminal Gasserian target (TGT) (13, 14) (**Figure 2**). RFT parameters: patients with V1 division involvement received continuous pulsed radiofrequency at 65 °C for one cycle of 60 seconds; V2 division involvement received 70 °C for two cycles of 120 seconds each; V3 division involvement received 75 °C for 2-4 cycles of 120 seconds each.

### Data analysis

Data were acquired on a 3.0 T Siemens Skyra (32-channel head coil). T1 MP-RAGE: TR/TE/TI 2000/2.29/900 ms, flip 8°, 0.9-mm isotropic. rs-fMRI: T2*-EPI, TR/TE 2000/30 ms, flip 90°, FOV 200×200 mm, 60 slices, 2.0/0.4 mm thickness/gap, 80×80, multiband 3, 10 min (300 volumes). Preprocessing (DPABI/MATLAB): discard first 10 volumes, slice-timing, realignment; exclude translation >2 mm, rotation >2°, or mean FD >0.3 mm (20). Coregister to T1; nuisance regression (6 motion, WM/CSF), detrend, band-pass 0.01–0.08 Hz. ReHo was computed in native space (3×3×3 voxels, Kendall’s W), global-mean normalized, then normalized to MNI with DARTEL, resampled to 2 mm, and smoothed (4-mm FWHM). Frames with FD >0.5 mm were scrubbed (proportion recorded). Cases with poor brain extraction/segmentation were excluded (21).

### Statistical analysis

Group-level analyses used a mass-univariate GLM with age, sex, and mean FD as covariates. Multiple-comparison control used permutation testing (5,000) with TFCE (two-tailed, FWE-corrected p<0.05; minimum cluster extent reported). Individual normative deviation maps were obtained by z-scoring each patient’s ReHo against age/sex-matched HCs, applying |Z|≥2.3 and cluster-wise FWE via permutation. For predicting ≤6-month recurrence, we fit penalized logistic regression with H-fMRI indicators (presence/side, V1 involvement) and clinical covariates (age, sex, disease duration, side, mean FD). Performance was optimism-corrected (0.632+ bootstrap, 1,000 resamples) with 95% CIs for AUC, sensitivity, specificity, and PPV/NPV.

### Clinical outcome assessment

Efficacy was evaluated at postoperative days 2–3 and at 6 months, 1 year, 2 years, and 3 years. Recurrence was graded by medication required to control pain (pre-specified standardized dose units): mild, < 50% of the preoperative dose with no additional intervention; moderate, 50–100%; severe, > 100% or uncontrolled pain requiring re-intervention. The primary endpoint for short-term recurrence was ≤ 6 months. “Effective” was defined as pain-free without medication or only mild recurrence; “Ineffective” as moderate or severe recurrence.

## Results

### Demographic and clinical data

Thirty TN patients were included: 15 managed with medication and 15 treated with PSR-DTI surgery. Full clinical characteristics are presented in **Table 1**.

**Table 1:** Characteristics of the total TN cohort (n=30) vs. PSR-DTI surgical subgroup (n=15).

Characteristic	Number of patients	Number of PSR-DTI patients
Total	30	15
Age and sex		
Age range	38-85	38-85
Age (Mean ± SD)	62.9 ± 11.5	65.5 ± 11.7
Male	13	6
Female	17	9
Affected side		
Right	18	11
Left	12	4
V1	1	1
V2	15	4
V3	3	1
V1 + V2	5	4
V2 + V3	3	2
V1 + V2 + V3	3	3

### Treatment outcomes

All 15 patients achieved successful TGT puncture. Sensory electrophysiological validation at the TGT (50 Hz, 1ms pulse) yielded voltage values ranging from 0.15-0.30 V.

Postoperative Days 2-3: 13 patients had VAS scores of 1-3, and two patients achieved VAS = 1 on postoperative days 4 and 14, respectively. Immediate overall efficacy was 86.6% (13/15).

6 Months Postoperative: 11 patients experienced complete pain relief, and four patients developed moderate or severe recurrence. Ultra-short-term efficacy was 73.3% (11/15); recurrence rate was 26.7% (4/15).

1 Year Postoperative: Effective treatment in 12 patients (including 11 with complete relief and 1 transitioning from moderate to mild recurrence). Ineffective treatment occurred in 3 patients. Short-term efficacy was 80% (12/15).

2 Years Postoperative: No new recurrences occurred. Long-term efficacy remained 80% (12/15).

3 Years Postoperative: One new moderate recurrence occurred (No.1). Long-term efficacy at 3 years was 73.3% (11/15).

Most recurrences (80%, 4/5) occurred within 6 months postoperatively. One recurrence (20%, 1/5) occurred at 3 years. No new recurrences arose between 6 months and 2 years. These findings indicate that precise PSR-DTI targeting of the TGT substantially inhibited (91%, 10/11) neural regeneration-mediated recurrences occurring between 1 year to 3 years.

### Differential brain region findings in TN subgroups

**Figure 3:**
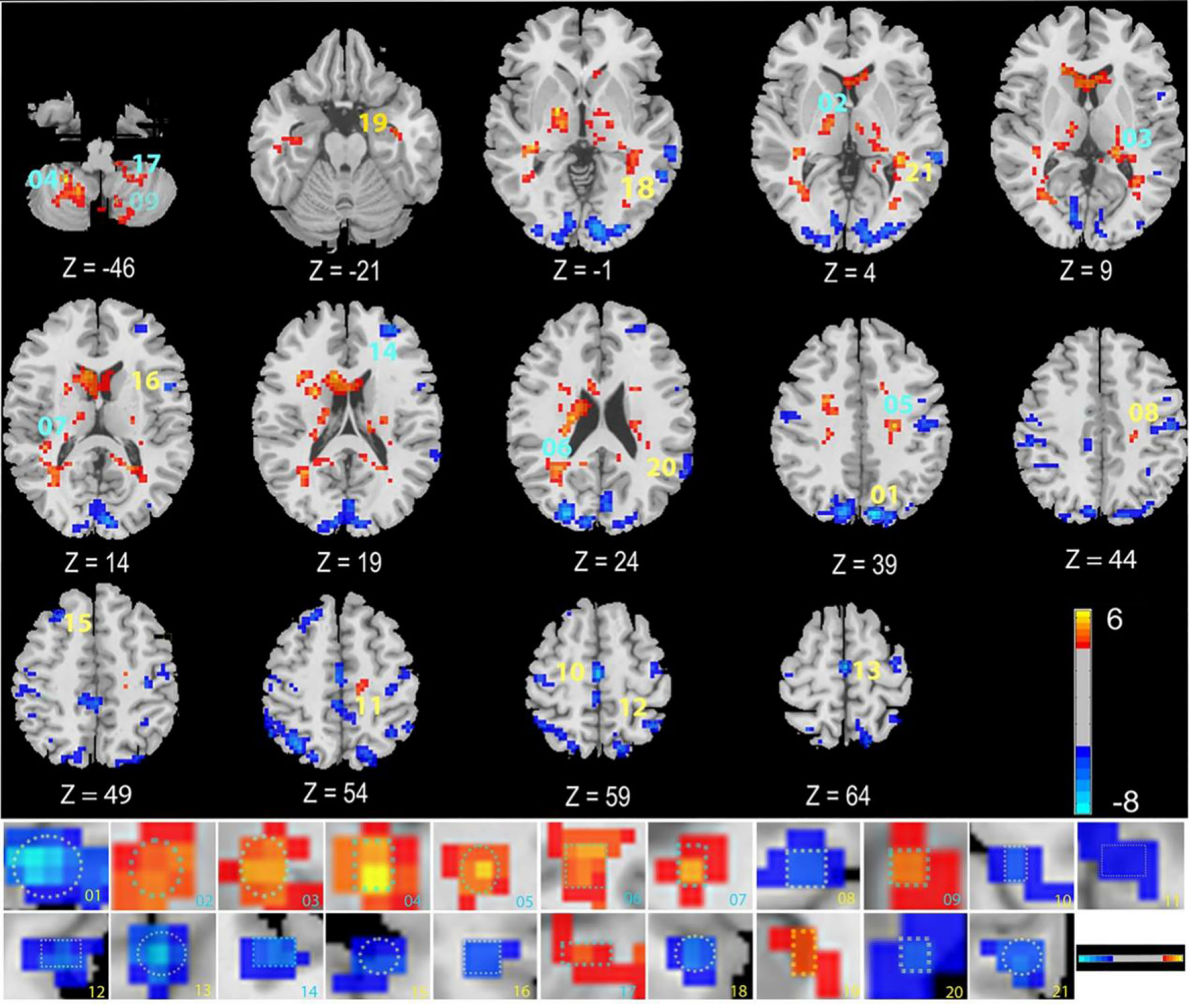
Regional homogeneity differences in TN patients. Differential analysis between TN patients and HC was performed using two-sample t-tests. The color bar represents the t-statistic values; warm colors (e.g., red/yellow) indicate brain regions with significantly increased ReHo values (positive t-values) in TN patients compared to controls, whereas cool colors (e.g., blue) indicate regions with significantly decreased ReHo values (negative t-values). The statistical significance threshold was set at GRF-corrected p < 0.05. Abbreviations: ReHo: Regional Homogeneity; TN: Trigeminal Neuralgia; HC: Healthy Controls; GRF: Gaussian Random Field.

TN Group: rs-fMRI identified 19 differential brain regions across all 30 TN patients (VAS 4-10) (**Figure 3**, **Table 2**). The presence of these regions may contribute to TN pathophysiology.

**Table 2:** T-test data for abnormal brain regions in TN patients vs. HC.

ALL no.	No.	Brain region	Cluster size (Voxels)	MNI (X Y Z)	T value
45	1	Cuneus_L	1367	-9 -81 39	-8.98862
76	2	Pallidum_R	575	18 -3 -3	6.12437
77	3	Thalamus_L	302	-21 -30 6	6.19444
104	4	Cerebellum_8_R	218	24 -48 -45	5.78382
33	5	Cingulum_Mid_L	192	-24 -18 39	5.58191
44	6	Calcarine_R	181	33 -51 24	5.14981
82	7	Temporal_Sup_R	157	39 -33 12	6.00197
57	8	Postcentral_L	120	-51 -18 42	-5.84031
103	9	Cerebellum_8_L	98	-12 -63 -48	4.88525
2	10	Precentral_R	94	42 -21 60	-4.92064
33	11	Cingulum_Mid_L	88	0 -36 51	-7.14651
61	12	Parietal_Inf_L	60	-48 -51 57	-6.04622
19	13	Supp_Motor_Area_L	56	0 -18 60	-7.35853
7	14	Frontal_Mid_L	53	-30 48 21	-5.85342
4	15	Frontal_Sup_R	51	24 27 51	-5.41887
17	16	Rolandic_Oper_L	46	-51 6 12	-5.73314
105	17	Cerebellum_9_L	40	-21 -45 -51	5.28106
85	18	Temporal_Mid_L	33	-57 -51 0	-6.1812
89	19	Temporal_Inf_L	32	-36 -6 -24	4.79442
81	20	Temporal_Sup_L	32	-63 -42 21	-4.87135
85	21	Temporal_Mid_L	31	-63 -36 3	-5.65972

AAL (Anatomical Automatic Labeling) parcellates the brain into 116 regions: 90 cerebral, 26 cerebellar. Regions listed: Calcarine_R, Temporal_Sup_R, Postcentral_L, Cerebellum_8_L, Precentral_R, Cingulum_Mid_L (X=-0, Y=-36, Z = 51), Parietal_Inf_L, Supp_Motor_Area_L, Frontal_Mid_L, Frontal_Sup_R, Rolandic_Oper_L, Cerebellum_9_L, Temporal_Mid_L (X=-57, Y=-51, Z = 0), Temporal_Inf_L, Temporal_Sup_L, Temporal_Mid_L (X=-63, Y=-36, Z = 3).

**Figure 4:**
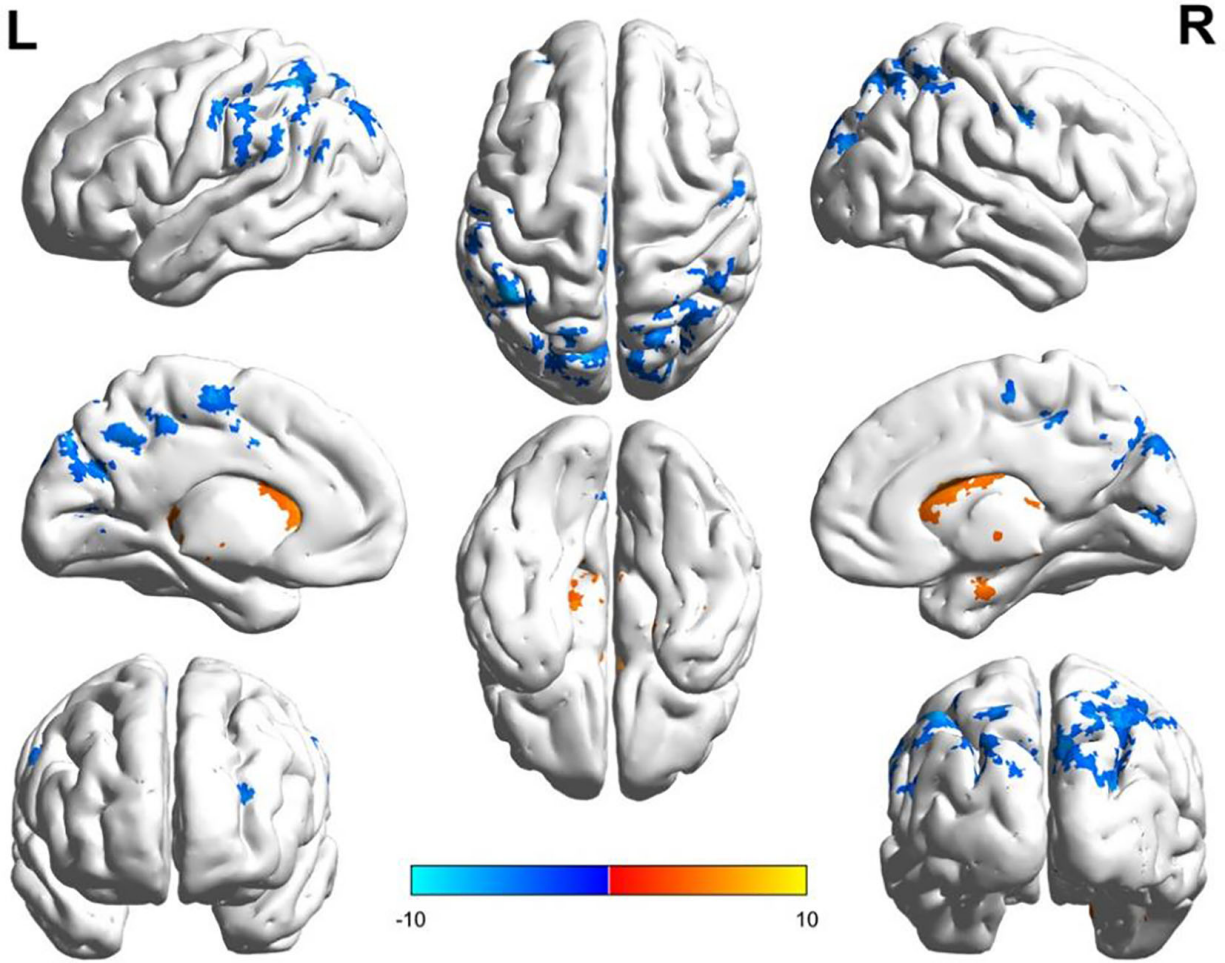
ReHo differences in PSR-DTI surgical patients. Differential analysis between the PSR-DTI group and HC was performed using two-sample t-tests, with results projected onto the brain surface. The color bar indicates the range of t-values: red/yellow regions denote significantly increased ReHo (positive t-values) relative to HC, while blue regions denote significantly decreased ReHo (negative t-values). The statistical threshold was set at GRF-corrected p < 0.05. Numbered regions correspond to: 1-Caudate_R, 2-Occipital_Sup_R, 3-Cerebellum_9_L, 4-Cerebellum_8_R, 5-Calcarine_L (X=-30, Y=-66, Z = 6), 6-Parietal_Inf_L, 7-Precuneus_L, 8-Postcentral_L, 9-Calcarine_L (X=-33, Y=-48, Z = 18), 10-Precuneus_R, 11-Supp_Motor_Area_L, 12-Temporal_Inf_L, 13-ParaHippocampal_R, 14-Postcentral_R, 15-Frontal_Mid_L, 16-Lingual_R, 17-SupraMarginal_L, 18-Frontal_Mid_L (X=-27, Y=-3, Z = 51).

PSR-DTI Group: Preoperative rs-fMRI identified 16 differential brain regions in the 15 PSR-DTI patients (VAS = 10) (**Figure 4**, **Table 3**). Compared to the TN group, 9 regions were novel: Caudate_R, Occipital_Sup_R, Calcarine_L, Precuneus_L, Precuneus_R, ParaHippocampal_R, Postcentral_R, Lingual_R, SupraMarginal_L. This suggests novel regional abnormalities may indicate progression requiring surgery.

**Table 3:** t-test data for abnormal brain regions in PSR-DTI patients vs. HC.

ALL no.	No.	Brain region	Cluster size (Voxels)	MNI (X Y Z)	T value
72	1	Caudate_R	1999	24 -24 42	8.25937
50	2	Occipital_Sup_R	663	27 -84 24	-7.77502
105	3	Cerebellum_9_L	316	-12 -51 -39	7.39727
104	4	Cerebellum_8_R	175	30 -63 -51	6.19134
43	5	Calcarine_L	174	-30 -66 6	5.99316
61	6	Parietal_Inf_L	135	-48 -51 57	-8.37313
67	7	Precuneus_L	118	-3 -54 42	-6.21712
57	8	Postcentral_L	105	-51 -15 45	-7.70574
43	9	Calcarine_L	65	-9 -90 0	-5.60083
68	10	Precuneus_R	60	18 -45 18	5.36338
19	11	Supp_Motor_Area_L	60	0 -15 63	-6.65415
89	12	Temporal_Inf_L	56	-39 -12 -18	5.69072
40	13	ParaHippocampal_R	53	24 -3 -27	5.58155
58	14	Postcentral_R	53	54 -6 39	-6.7594
7	15	Frontal_Mid_L	52	-33 48 18	-8.17244
48	16	Lingual_R	50	12 -75 -6	-7.52571
63	17	SupraMarginal_L	33	-63 -45 30	-4.91563
7	18	Frontal_Mid_L	30	-27 -3 51	-5.55443

Rs-fMRI in the 4 recurrence patients identified 15 differential regions, including: Insula, Fusiform_L, Vermis_3, Cerebellum_3_R, Temporal_Sup_L, Fusiform_R, Caudate_L, Calcarine_L, Cerebellum_Crus2_R, Temporal_Mid_R, Lingual_R, Hippocampus_L, Precuneus_L, Cerebellum_8_L and Thalamus_R. These regions (we named them as 1-15, respectively) constitute a library of potential predictive brain regions (PPBR) for recurrence.

### Screening predictive brain regions for short-term recurrence

**Figure 5:**
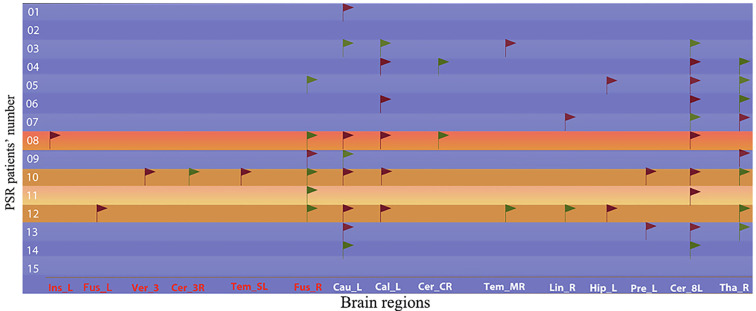
Positive sites of potential predictive brain regions for recurrence in TN patients. Treatment Outcome: =Excellent (Pain-free, no meds); =Good (Mild recurrence; pain reduction ≥50%, reduced meds); =Fair (Slight relief; pain reduction <50%); =Poor (No relief). Laterality: =Ipsilateral; =Contralateral.

Entire PSR-DTI Group (n=15): Total PPBR occurrences were 53 instances across all patients which including 13 patients exhibited 1-9 PPBRs and 2 patients had no PPBR, 45% in ipsilateral and other 55% in contralateral (**Figure 5**).

PSR-DTI Recurrence Subgroup (n=4): Total PPBRs were 25 instances, mean times was 6.3 in per patient, 40% in ipsilateral and 60% in contralateral.

PSR-DTI Non-Recurrence Subgroup (n=11): Total PPBRs were 28 instances, mean times was 2.5 in per patient, 61% in ipsilateral and 39% in contralateral.

Patients with recurrence exhibited a significantly higher mean PPBR burden (6.3 vs. 2.5; mean difference 2.5-fold, 95% CI: 2.1-5.5; t (13) = 3.92, *p* = 0.002, Cohen**’**s d = 1.74) and a greater proportion of contralateral PPBRs (61% vs. 39%) compared to non-recurrence patients. These findings indicate that both novel and contralateral abnormalities are associated with an increased risk of recurrence.

### Preliminary screening of predictive regions

**Figure 6:**
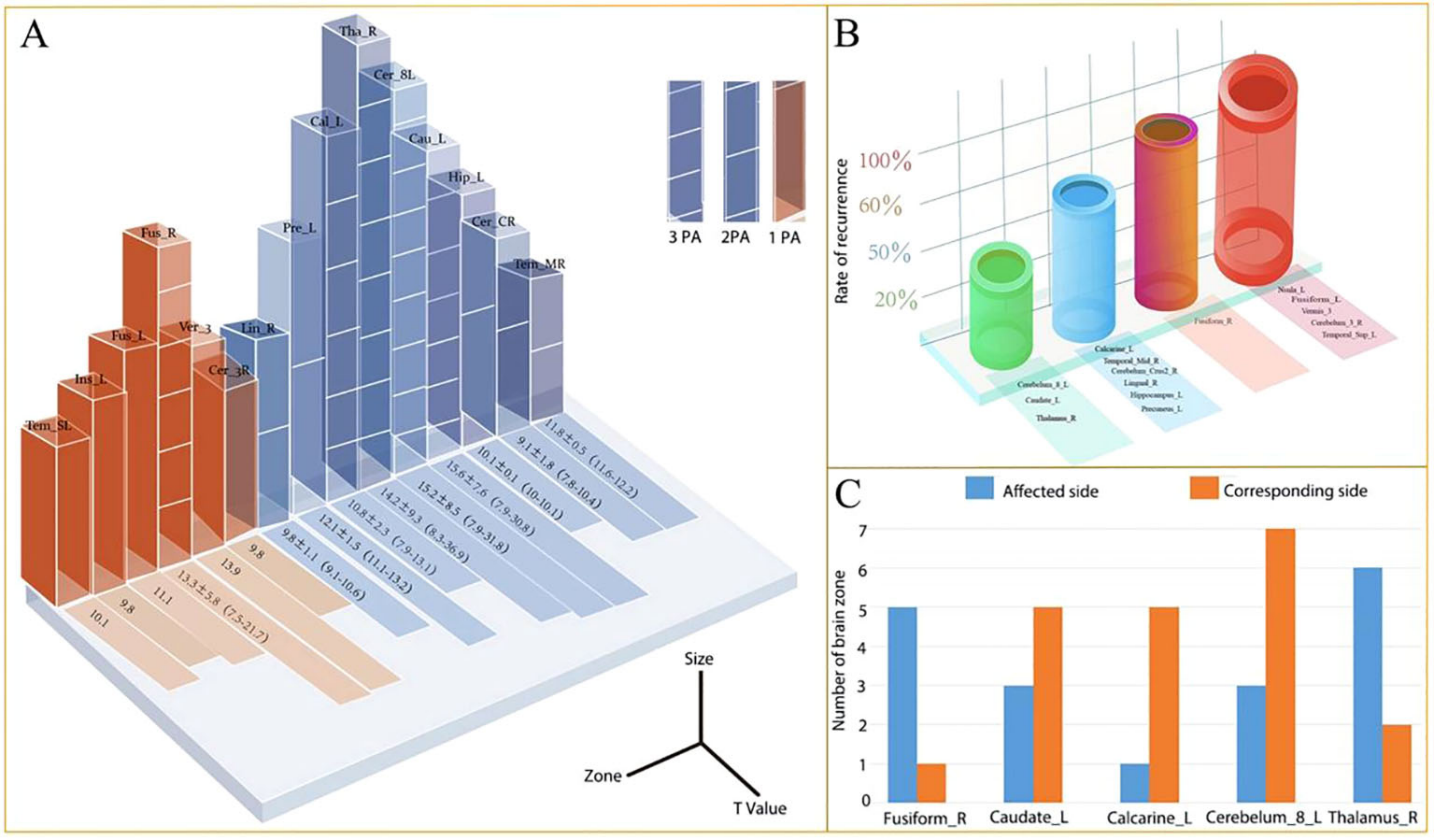
Characteristics of potential predictive brain regions for pain recurrence in PSR-DTI patients. **(A)** Number and value of positive brain regions. **(B)** Recurrence rate per PPBR. **(C)** Distribution of common abnormal brain regions (3 PA = 3 patients; 2 PA = 2 patients; 1 PA = 1 patient).

Frequency & Recurrence Rate: Regions 1-5, Occurred only once, and exclusively in recurrence patients (100% recurrence rate); Regions 6-15, Occurred in both recurrence and non-recurrence patients; Regions 6, 7, 8, 14, 15 (common, 6-10 occurrences) and 9-13 (rare, all were 2 occurrences) showed varying recurrence rates (**Figure 6**, **Table 4**). Thus, regions 1-5 were selected as primary predictive candidates, and Region 6 (Fusiform_R) was identified as a secondary candidate due to its relatively higher recurrence rate.

**Table 4:** Complete remission and recurrence rates for patients stratified by potential recurrence-related brain regions.

Brain zone	Quantity of appearance	Complete remission and percentage (%)	Recurrence and percentage (%)
Insula-L	1	0	1(100%, 1/1)
Fusiform_L	1	0	1(100%, 1/1)
Vermis_3	1	0	1(100%, 1/1)
Cerebellum_3_R	1	0	1(100%, 1/1)
Temporal_Sup_L	1	0	1(100%, 1/1)
Fusiform_R	6	2(33%, 2/6)	4(67%, 4/6)
Caudate_L	8	5 (63%, 5/8)	3(37%, 3/8)
Calcarine_L	6	3(50%, 3/6)	3(50%, 3/6)
Cerebellum_Crus2_R	2	1(50%, 1/2)	1(50%, 1/2)
Temporal_Mid_R	2	1(50%, 1/2)	1(50%, 1/2)
Lingual_R	2	1(50%, 1/2)	1(50%, 1/2)
Hippocampus_L	2	1(50%, 1/2)	1(50%, 1/2)
Precuneus_L	2	1(50%, 1/2)	1(50%, 1/2)
Cerebellum_8_L	10	7(70%, 7/10)	3(30%, 3/10)
Thalamus_R	8	6(75%, 6/8)	2(25%, 2/8)

Red text indicates brain regions with the highest recurrence rates, and blue text indicates regions with the second-highest recurrence rates.

### Refining the predictive role of Fusiform_R

**Figure 7:**
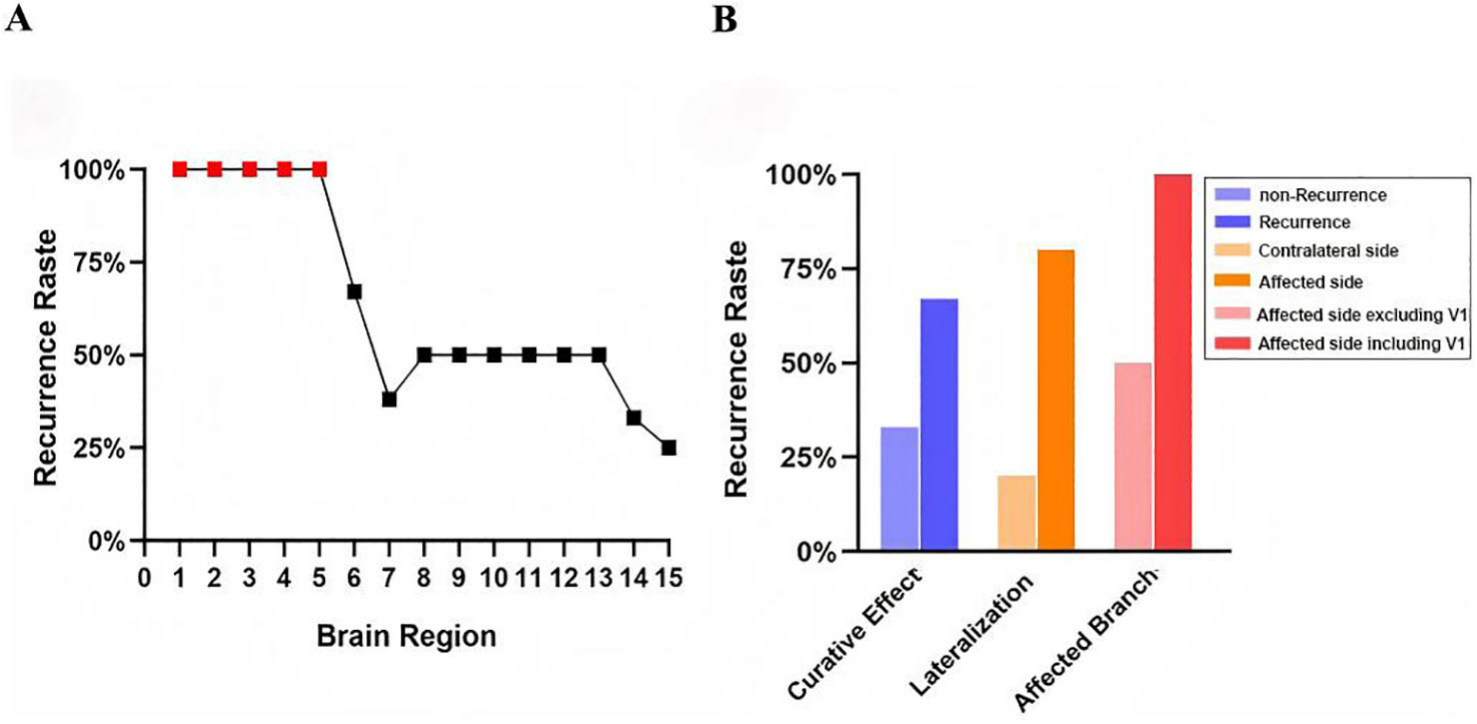
Recurrence risk stratification based on fMRI-Defined brain regions. **(A)**Recurrence rates across 15 potential predictive brain regions. **(B)** Differential recurrence rates associated with Fusiform_R under specific clinical conditions.

Overall Recurrence Rate: 67% (4/6) (**Figure 7A**). Laterality: recurrence rate was 80% (4/5) for ipsilateral Region 6 abnormality vs. 0% (0/1) for contralateral. Trigeminal branch involvement: recurrence rate was 100% (2/2) for patients with region 6 abnormality and V1 division pain (Case 10: V1+V2+V3; Case 12: V1+V2), recurrence rate was 50% (2/4) for region 6 abnormality without V1 pain (**Figure 7B**). Thus, ipsilateral region 6 abnormality combined with V1 pain predicted recurrence with 100% accuracy, equivalent to regions 1-5.

### Recurrence risk stratification via final predictive brain regions

**Figure 8:**
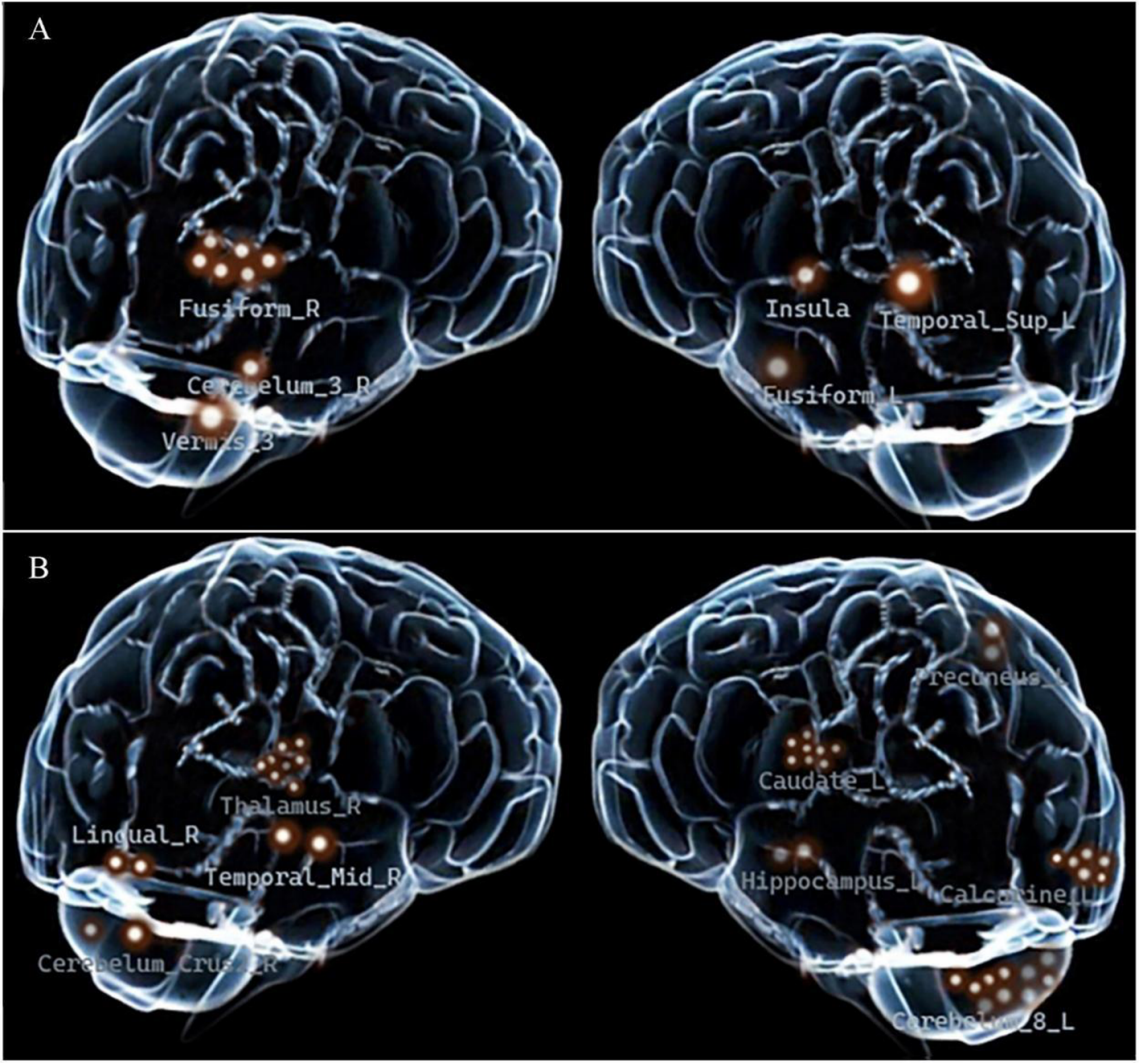
Spatial distribution of fMRI-based risk regions in surgical trigeminal neuralgia patients. **(A)** Six kinds of high-risk fMRI regions. **(B)** Nine middle- and low-risk fMRI regions. Each highlighted marker represents a positive finding in a single patient scan.

Recurrence rates for unilateral Regions 7-15 ranged from 33%-60% (**Table 5**). Six regions with 100% recurrence rate within contralateral Insula, Fusiform_L, Vermis_3 and Temporal_Sup_L, ipsilateral Cerebellum_3_R, Fusiform_R with V1 division pain, constitute the final set of High-Risk fMRI (H-fMRI) regions of recurrence (**Figure 8**).

**Table 5:** Treatment outcomes by laterality (ipsilateral vs. contralateral) for each potential recurrence-related brain region in patients.

Brain zone	Affected side			Contralateral side		
	Number of brain regions	Number of good effects	Number and percentage of poor effects (%)	Number of brain regions	Number of good effects	Number and percentage of poor effects (%)
Insula-L	0	0	0	1	0	1(100%, 1/1)
Fusiform_L	0	0	0	1	0	1(100%, 1/1)
Vermis_3	0	0	0	1	0	1(100%, 1/1)
Cerebellum_3_R	1	0	1(100%, 1/1)	0	0	0
Temporal_Sup_L	0	1	0	1	0	1(100%, 1/1)
Fusiform_R	5	1	4(80%, 4/5)	1	1	0
Caudate_L	3	3	0	5	2	3(60%, 3/5)
Calcarine_L	1	1	0	5	2	3(60%, 3/5)
Cerebellum_Crus2_R	2	1	1(50%, 1/2)	0	0	0
Temporal_Mid_R	1	0	1(50%, 1/2)	1	1	0
Lingual_R	1	0	1(50%, 1/2)	1	1	0
Hippocampus_L	0	0	0	2	1	1(50%, 1/2)
Precuneus_L	0	0	0	2	1	1(50%, 1/2)
Cerebellum_8_L	3	3	0	7	4	3(43%, 3/7)
Thalamus_R	6	4	2(33%, 2/6)	2	2	0

**Figure 9:**
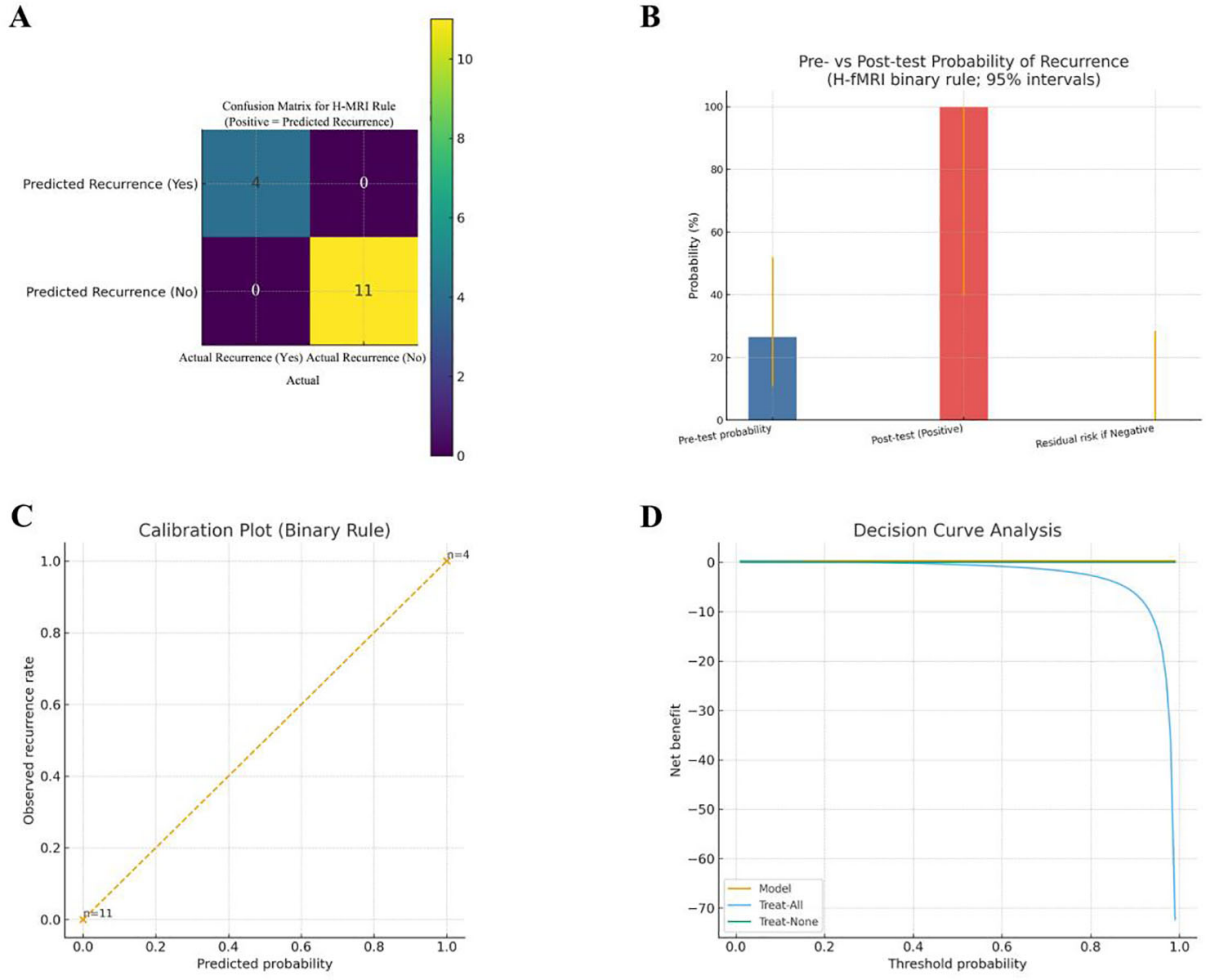
Apparent performance of the pre-specified H-fMRI rule in the PSR-DTI derivation cohort (n=15; 4 recurrences ≤6 months). **(A)** Confusion matrix (positive = predicted recurrence): TP = 4, TN = 11, FP = 0, FN = 0, showing apparent perfect classification. **(B)** Pre-/post-test probability of recurrence (4/15, 4/4, 0/11; 95% CIs). **(C)** Calibration: two-point calibration for the binary rule (observed recurrence rates at predicted = 1 and predicted = 0); the 45° line denotes ideal calibration. **(D)** Decision curve: net benefit across threshold probabilities 0.01–0.99; the model overall outperforms Treat-All and Treat-None.

The pre-specified six-region H-fMRI binary rule achieved apparent perfect discrimination in the PSR-DTI derivation cohort and showed marked post-test probability shifts with favorable net benefit on decision-curve analysis (**Figure 9**).

### Analysis of H-fMRI regional location characteristics

**Figure 10:**
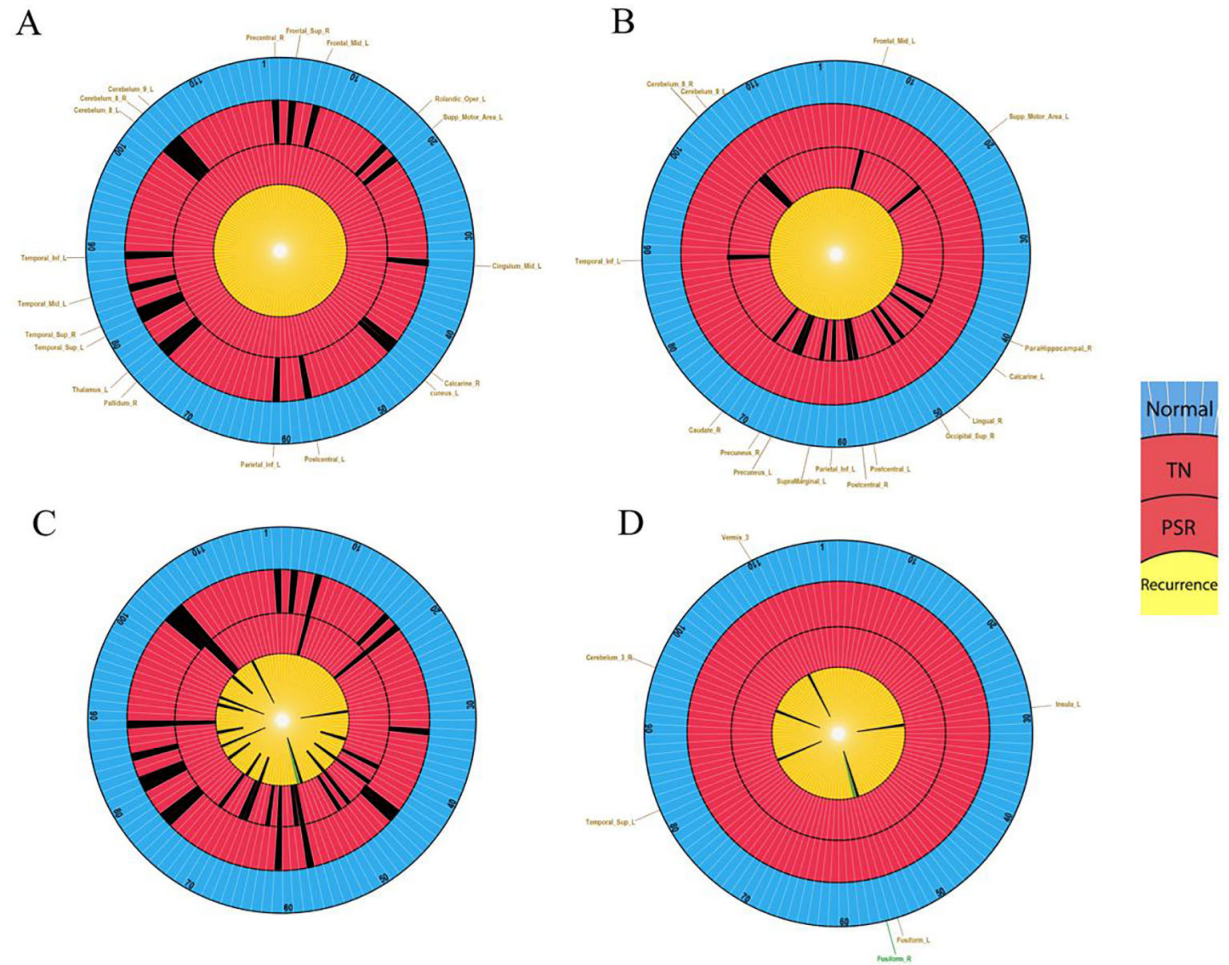
Schematic representation of brain region locations in healthy individuals and TN patients. **(A)** Healthy individuals (116 regions, blue circle) vs. TN patients VAS 4-10 (19 positive regions, outer red circle). **(B)** PSR-DTI patients VAS 8-10 (16 positive regions, inner red circle). **(C)** Recurrence patients (15 Potential Predictive Brain Regions, yellow circle). **(D)** Recurrence patients (6 high-risk fMRI Regions, yellow circle). Insets show regional distributions.

Automated Anatomical Labeling (AAL) Atlas includes 116 normal regions. TN Group (19 regions) vs. PSR-DTI Group (16 regions), 7 overlapping regions (36.8% overlap); recurrence PPBRs (15 regions) vs. PSR-DTI Group, 3 overlapping regions (18.7% overlap); H-fMRI Regions (6 regions) vs. PSR-DTI Group, 0% overlap; H-fMRI Regions vs. TN Group, 1 overlapping region (5.2% overlap) (**Figure 10**). These results suggesting that patient-specific H-fMRI regions may drive short-term recurrence.

### Changes in brain regions 2-3 days post-PSR-DTI

**Figure 11:**
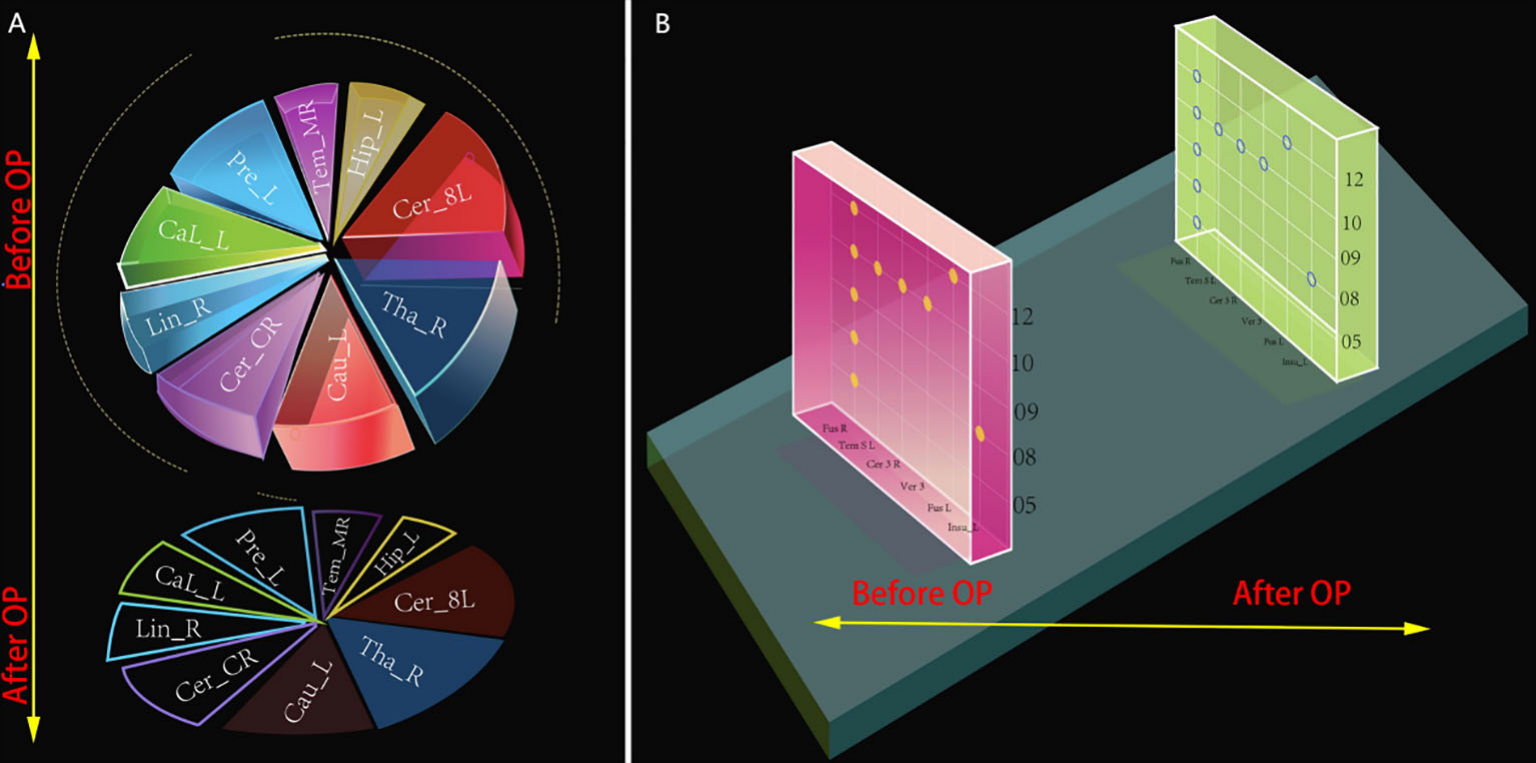
Schematic of potential predictive brain regions pre- and post PSR-DTI in TN patients. **(A)** Nine regions positive preoperatively in both outcome groups (Top). Six boxed regions disappeared postoperatively (Bottom); three unboxed regions showed weakened activity. **(B)** Five regions positive preoperatively only in the poor outcome group, plus Fusiform_R (Left). All six regions disappeared postoperatively (Right).

13 patients whom VAS were 1-3: All 15 recurrence-related differential regions disappeared (100% elimination). Two patients with VAS scores of 4: 12 recurrence-related regions (6 H-fMRI, 6 L-fMRI) disappeared (80%, 12/15), and 3 regions weakened (Caudate_L & Cerebellum_8_L in Case No.8; Thalamus_R in Case No.10) (**Figure 11**).

## Discussion

Recurrence in TN patients after RFT can be categorized into two types based on the timing of onset: short-term recurrence (STR) which occurring within 1 year postoperatively, and long-term recurrence (LTR) which occurring more than 1 year postoperatively. Reported rates of STR and LTR vary considerably in the literature. STR rates fall into three ranges: low range (<9.8%) (22–26), medium range (12%—17%) (27–30), and high range (21%—56%) (31–34). Two-year LTR rates are distributed into two ranges: low range (7%—17%) (25, 26, 35) and high range (20%—50%) (5, 28, 29, 33, 36, 37).

The currently prevailing theoretical explanation for LTR is the regeneration of nerve fibers within the TGT occurring 1-3 years postoperatively, leading to the reappearance of pain (5), termed neuroregeneration-mediated recurrence. Regarding STR, we propose that it represents non-neuroregeneration-mediated recurrence under the premise of effective RFT, i.e., recurrence due to patient-specific factors.

Indeed, not only STR research had been received considerable attention (31–34), but STR occurring even earlier (within 1-6 months) has also been noted (23, 24, 31, 38). J. Piquer et al. (39) reported a 1-year recurrence rate of 13% (13/98), but patients recurring within one year accounted for 46% (13/30) of all recurrences observed over more than three years. In our cohort, STR patients constituted 80% (4/5) of all recurrences within 3 years postoperatively. We speculate that the unusually high STR rate in our group may be related to a relatively higher proportion of patients with specific constitutional factors.

To date, 23 brain regions have been identified by various researchers as abnormally functional in TN patients (2, 40–42). We identified 19 abnormal functional brain regions in TN patients. The majority of these abnormal regions (68.4%, 13/19) were fully consistent with literature reports, a smaller portion (21.0%, 4/19) partially consistent, while abnormalities in the Rolandic_Oper_L and Cerebellum_9_L regions have not been previously reported in the literature.

Furthermore, in 15 PSR group patients with VAS scores of 10, we identified 16 abnormal brain regions. Among these, abnormalities in 9 regions related to pain perception, pain processing, emotional processing, vision, and memory differed from those observed in the broader TN group. This finding suggests that TN patients with severe pain possess unique characteristics in their abnormal brain region distribution. Among these 16 abnormal regions, abnormalities in Cerebellum_9_L, Lingual_R, and Calcarine_L have not been previously reported.

Overall, our study identified four novel abnormal brain regions in TN patients: Rolandic_Oper_L, Cerebellum_9_L, Lingual_R, and Calcarine_L. We attribute these findings to differences in the clinical characteristics of enrolled TN patients and variations in fMRI methodologies across studies. In fact, Liang Y et al. (43) also noted a lack of consistency in the locations of abnormal brain regions reported by different centers conducting fMRI research on TN patients.

Traditionally associated with motor control, the cerebellum is increasingly recognized for its critical role in non-motor functions, including pain modulation and emotional processing. The “cognitive-affective cerebellum” theory suggests that the posterior lobe and vermis serve as integral nodes within the pain matrix and salience network, specifically influencing the affective-motivational dimension of pain. Persistent abnormalities in these regions (e.g., Vermis_3 and Cerebellum_3_R) observed in our high-risk cohort may reflect entrenched central sensitization or maladaptive emotional learning associated with chronic neuropathic pain. Consequently, these central circuit disturbances could drive early symptom recurrence, independent of the technical success of the peripheral nerve intervention.

Departing from previous group-level comparison methods, this study also performed individual case-control comparisons for each of the 15 PSR group patients against a normal control group. Based on this cohort, all patients exhibiting abnormalities in one or more of the following regions (contralateral Insula_L, Fusiform_L, Vermis_3, Temporal_Sup_L, and ipsilateral Cerebellum_3_R, Fusiform_R with V1 involvement) experienced short-term recurrence after PSR-DTI. We therefore refer to these regions as candidate high-risk fMRI regions for recurrence risk stratification, pending external validation. Consequently, we define these 6 brain regions as high-risk recurrence regions on fMRI (H-fMRI) for individual TN patients undergoing PSR. Abnormalities in the Vermis_3 and Cerebellum_3_R regions in TN patients have not been previously reported.

The distinct predictive value of V1 division involvement combined with Fusiform_R abnormalities may be attributed to both anatomical and functional factors. Clinically, radiofrequency ablation of the ophthalmic (V1) division often requires more conservative temperature parameters to preserve the corneal reflex, which can potentially lead to less complete denervation compared to procedures targeting the V2 or V3 divisions. Functionally, the fusiform gyrus is integral to higher-order visual and object processing. The specific coupling of V1 pain—which involves the ophthalmic nerve—with fusiform abnormalities suggests a specific maladaptive neuroplasticity within visual-pain associative pathways. This unique central network alteration may be more resistant to standard thermal coagulation, thereby predisposing patients to higher rates of early recurrence.

In this study, PSR surgery resulted in the elimination or reduction of recurrence-related differential brain regions in each TN patient. This outcome aligns with Dou Z et al. (44) reporting decreased ReHo values in the Middle Temporal Gyrus, Postcentral Gyrus, and left Insula after RFT; matches Moisset X et al. (2)reporting the disappearance of Insula activation 1-2 months post-RFT; and is largely consistent with Wen-Ching Liu et al. (45) reporting significant regression of Insula and Cerebellar activation 1-2 weeks post-RRT. These findings demonstrate that PSR-DTI surgery effectively modulates abnormal brain region activity in TN patients.

Research on individualized rs-fMRI (irs-fMRI) is clinically essential for using abnormal brain regions as an indicator to predict postoperative recurrence risk. Theoretically, irs-fMRI results could be influenced by differences in patient state during pre- and postoperative scans. In this study, irs-fMRI examinations focusing on abnormal regions within the potential recurrence library confirmed that all 15 abnormal regions were either eliminated or attenuated following effective immediate treatment. This result suggests that targeting specific brain regions is an effective approach for individualized fMRI assessment.

There are reports that TN patients whom with increased ReHo values in Insula and/or Vermis experienced moderate short-term recurrence post-RFT (2, 44). Those reports had some extent corroborate our finding that Insula and Vermis might contribute to short-term recurrence after RFT. However, the other four of our fund 6 H-fMRI regions as causes of short-term recurrence not previously reported. For the highest recurrence rates (100%) of fMRI compare with MRI (5%-15%) and DTI (60%). Taken together, these observations suggest that abnormal functional brain regions may represent an important correlate of short-term recurrence risk after RFT/PSR-DTI in TN. However, the predictive utility and generalizability of these candidate regions require prospective evaluation in larger, independent cohorts.

This study has certain limitations. We applied lateralization and branch-specific restrictions to the H-fMRI regions in this timeframe. As research sample sizes increase in the future, these lateralization and branch restrictions for H-fMRI regions may require further refinement.

## Conclusion

Rolandic_Oper_L, Cerebellum_9_L, Lingual_R, and Calcarine_L were newly discovered abnormal brain regions in trigeminal neuralgia patients. For the first time, six high-risk recurrence regions on fMRI highly associated with short-term postoperative recurrence were identified: contralateral Insula_L, Fusiform_L, Vermis_3 and Temporal_Sup_L, ipsilateral Cerebellum_3_R, Fusiform_R (specifically associated with V1 division involvement). This study provides an objective imaging basis for individualized prediction of early recurrence risk after PSR-DTI in trigeminal neuralgia.

## Data Availability

The original contributions presented in the study are included in the article/supplementary material. Further inquiries can be directed to the corresponding authors.

## References

[1] Bendtsen L Zakrzewska JM Heinskou TB Hodaie M Leal PRL Nurmikko T . Advances in diagnosis, classification, pathophysiology, and management of trigeminal neuralgia. Lancet Neurol. (2020) 19:784–96. doi: 10.1016/S1474-4422(20)30233-7, PMID: 32822636

[2] Moisset X Villain N Ducreux D Serrie A Cunin G Valade D . Functional brain imaging of trigeminal neuralgia. Eur J Pain. (2011) 15:124–31. doi: 10.1016/j.ejpain.2010.06.006, PMID: 20609605

[3] Cruccu G Finnerup NB Jensen TS Scholz J Sindou M Svensson P . Trigeminal neuralgia: New classification and diagnostic grading for practice and research. Neurology. (2016) 87:220–8. doi: 10.1212/WNL.0000000000002840, PMID: 27306631 PMC4940067

[4] Cruccu G Di Stefano G Truini A . Trigeminal neuralgia. N Engl J Med. (2020) 383:754–62. doi: 10.1056/NEJMra1914484, PMID: 32813951

[5] Son BC Kim HS Kim IS Yang SH Lee SW . Percutaneous radiofrequency thermocoagulation under fluoroscopic image-guidance for idiopathic trigeminal neuralgia. J Korean Neurosurg Soc. (2011) 50:446–52. doi: 10.3340/jkns.2011.50.5.446, PMID: 22259692 PMC3259465

[6] Mousavi SH Gehling P Burchiel KJ . The long-term outcome of radiofrequency ablation in multiple sclerosis-related symptomatic trigeminal neuralgia. Neurosurgery. (2022) 90:293–9. doi: 10.1227/NEU.0000000000001817, PMID: 35113822

[7] Gunduz HB Cevik OM Asilturk M Gunes M Uysal ML Sofuoglu OE . Percutaneous radiofrequency thermocoagulation in trigeminal neuralgia: analysis of early and late outcomes of 156 cases and 209 interventions. J Korean Neurosurg Soc. (2021) 64:827–36. doi: 10.3340/jkns.2020.0333, PMID: 34320779 PMC8435657

[8] Jain A . Comparative analysis of balloon compression and radiofrequency ablation in idiopathic trigeminal neuralgia: A retrospective study with a 24-month follow-up. Turk J Anaesthesiol Reanim. (2019) 47:146–50. doi: 10.5152/TJAR.2019.53533, PMID: 31080957 PMC6499048

[9] Elawamy A Abdalla EEM Shehata GA . Effects of pulsed versus conventional versus combined radiofrequency for the treatment of trigeminal neuralgia: A prospective study. Pain Physician. (2017) 20:E873–e881., PMID: 28934792

[10] Cohen SP Mao J . Neuropathic pain: mechanisms and their clinical implications. Bmj. (2014) 348:f7656. doi: 10.1136/bmj.f7656, PMID: 24500412

[11] Zheng JH Sun K Zhang HT Xie YJ Wang-Yang LX Chen HY . A study on the recurrence rate of trigeminal neuralgia after MVD and the related factors. J Neurol Surg B Skull Base. (2020) 81:572–8. doi: 10.1055/s-0039-1692687, PMID: 33134025 PMC7591360

[12] Sarsam Z Garcia-Fiñana M Nurmikko TJ Varma TR Eldridge P . The long-term outcome of microvascular decompression for trigeminal neuralgia. Br J Neurosurg. (2010) 24:18–25. doi: 10.3109/02688690903370289, PMID: 20158348

[13] Ma ZS Su X Wang ZM Wang ZJ Cheng M Tian Y . A novel potential measurement indicator with objective and quantitative effect for trigeminal neuralgia: fractional anisotropy in MR-DTI. Front Neurol. (2024) 15. doi: 10.3389/fneur.2024.1453431, PMID: 39777313 PMC11703729

[14] Su X Wang Z Wang Z Cheng M Du C Tian Y . A novel indicator to predict the outcome of percutaneous stereotactic radiofrequency rhizotomy for trigeminal neuralgia patients: diffusivity metrics of MR-DTI. Sci Rep. (2024) 14:9235. doi: 10.1038/s41598-024-59828-4, PMID: 38649718 PMC11035693

[15] Logothetis NK . What we can do and what we cannot do with fMRI. Nature. (2008) 453:869–78. doi: 10.1038/nature06976, PMID: 18548064

[16] Li L Du H Li XY Yu CM Huang BB Ma ZT . A study of brain function changes in patients with trigeminal neuralgia of different laterality based on rs-fMRI. J Oral Facial Pain Headache. (2025) 39:148–56. doi: 10.22514/jofph.2025.015, PMID: 40129433 PMC11934743

[17] Deng X Liu L Chen J Liu Z Feng H . Cognitive decline in patients with trigeminal neuralgia: A resting-state fMRI study. Brain Behav. (2025) 15:e70434. doi: 10.1002/brb3.70434, PMID: 40135636 PMC11938110

[18] Wang Z Zhao Z Song Z Xu J Wang Y Zhao Z . Functional alterations of the brain default mode network and somatosensory system in trigeminal neuralgia. Sci Rep. (2024) 14:10205. doi: 10.1038/s41598-024-60273-6, PMID: 38702383 PMC11068897

[19] Latorre G González-García N García-Ull J González-Oria C Porta-Etessam J Molina FJ . Diagnosis and treatment of trigeminal neuralgia: Consensus statement from the Spanish Society of Neurology’s Headache Study Group. Neurología (English Edition). (2023) 38:S37–52. doi: 10.1016/j.nrleng.2023.04.005, PMID: 37116695

[20] Yan CG Wang XD Zuo XN Zang YF . DPABI: data processing & Analysis for (Resting-state) brain imaging. Neuroinformatics. (2016) 14:339–51. doi: 10.1007/s12021-016-9299-4, PMID: 27075850

[21] Jiang L Zuo XN . Regional homogeneity: A multimodal, multiscale neuroimaging marker of the human connectome. Neuroscientist. (2016) 22:486–505. doi: 10.1177/1073858415595004, PMID: 26170004 PMC5021216

[22] Zheng S Yuan R Ni J Liu H Yang Y Zhang S . Long-term recurrence-free survival and complications of percutaneous balloon compression and radiofrequency thermocoagulation of Gasserian ganglion for trigeminal neuralgia: A retrospective study of 1313 cases. Pain Pract. (2022) 22:532–40. doi: 10.1111/papr.13114, PMID: 35460524

[23] Wang Q Du WJ . Analysis of short-term efficacy of radiofrequency thermocoagulation in the treatment of classic trigeminal neuralgia. Agri. (2022) 34:1–6. doi: 10.14744/agri.2021.42800, PMID: 34988955

[24] Liu G Du Y Wang X Ren Y . Efficacy and safety of repeated percutaneous radiofrequency thermocoagulation for recurrent trigeminal neuralgia. Front Neurol. (2018) 9:1189. doi: 10.3389/fneur.2018.01189, PMID: 30713521 PMC6345700

[25] Yu D Xie K . A predictive model for the risk of postsurgery pain recurrence in the V1 branch of the trigeminal nerve. Pain Physician. (2024) 27:E147–e155. doi: 10.36076/ppj.2024.27.e147, PMID: 38285046

[26] Ran B Wei J Zhong Q Fu M Yang J Chen X . Long-term follow-up of patients treated with percutaneous radiofrequency thermocoagulation via the foramen rotundum for isolated maxillary nerve idiopathic trigeminal neuralgia. Pain Med. (2019) 20:1370–8. doi: 10.1093/pm/pnz006, PMID: 30835786

[27] Li X Zheng S Cao Z He L Yang L Ni J . Factors associated with long-term risk of recurrence after percutaneous radiofrequency thermocoagulation of the gasserian ganglion for patients with trigeminal neuralgia involving the ophthalmic division: A retrospective study. Pain Pract. (2021) 21:26–36. doi: 10.1111/papr.12930, PMID: 32585754

[28] Zheng S Li X Li R Yang L He L Cao G . Factors associated with long-term risk of recurrence after percutaneous radiofrequency thermocoagulation of the gasserian ganglion for patients with trigeminal neuralgia: A multicenter retrospective analysis. Clin J Pain. (2019) 35:958–66. doi: 10.1097/AJP.0000000000000758, PMID: 31490204

[29] Zhao W Yang L Deng A Chen Z He L . Long-term outcomes and predictors of percutaneous radiofrequency thermocoagulation of Gasserian ganglion for maxillary trigeminal neuralgia: a retrospective analysis of 1070 patients with minimum 2-year follow-up. Ann Med. (2022) 54:2420–30. doi: 10.1080/07853890.2022.2117409, PMID: 36148904 PMC9518273

[30] Xi L Liu X Shi H Han W Gao L Wang L . Comparative safety and efficacy of percutaneous radiofrequency thermocoagulation and percutaneous balloon compression in CT-guided and local anesthesia for recurrent trigeminal neuralgia. Front Neurol. (2023) 14:1336261. doi: 10.3389/fneur.2023.1336261, PMID: 38249730 PMC10797886

[31] Chang KW Jung HH Chang JW . Percutaneous procedures for trigeminal neuralgia. J Korean Neurosurg Soc. (2022) 65:622–32. doi: 10.3340/jkns.2022.0074, PMID: 35678088 PMC9452389

[32] Herta J Loidl TB Schmied T Tomschik M Khalaveh F Wang WT . Retrospective comparison of percutaneous balloon compression and radiofrequency-thermocoagulation in the management of trigeminal neuralgia. Acta Neurochir (Wien). (2023) 165:1943–54. doi: 10.1007/s00701-023-05656-w, PMID: 37286804 PMC10319675

[33] Udupi BP Chouhan RS Dash HH Bithal PK Prabhakar H . Comparative evaluation of percutaneous retrogasserian glycerol rhizolysis and radiofrequency thermocoagulation techniques in the management of trigeminal neuralgia. Neurosurgery. (2012) 70:407–12; discussion 412–3. doi: 10.1227/NEU.0b013e318233a85f, PMID: 21866065

[34] Noorani I Lodge A Vajramani G Sparrow O . Comparing percutaneous treatments of trigeminal neuralgia: 19 years of experience in a single centre. Stereotact Funct Neurosurg. (2016) 94:75–85. doi: 10.1159/000445077, PMID: 27071078

[35] Taha JM Tew JM Jr. Buncher CR . A prospective 15-year follow up of 154 consecutive patients with trigeminal neuralgia treated by percutaneous stereotactic radiofrequency thermal rhizotomy. J Neurosurg. (1995) 83:989–93. doi: 10.3171/jns.1995.83.6.0989, PMID: 7490643

[36] Deng J Fei Y Huang B . Nomogram for predicting the recurrence rate in selective radiofrequency thermocoagulation of the trigeminal nerve based on regression via least absolute shrinkage and selection operator. Pain Physician. (2023) 26:E341–e352. doi: 10.36076/ppj.2023.26.E341, PMID: 37535781

[37] Wu F Mei Z Xie K . Risk factors for recurrence after radiofrequency surgery of the V2 branch of the trigeminal nerve. Pain Physician. (2023) 26:E601–e609. doi: 10.36076/ppj.2023.26.e601, PMID: 37774198

[38] Li Y Guo Y Yang L Ni J . Comparison of the short-term outcomes after low-temperature plasma radiofrequency ablation (coblation) in the Gasserian ganglion for the treatment of idiopathic trigeminal neuralgia. J Pain Res. (2019) 12:1235–42. doi: 10.2147/JPR.S199504, PMID: 31114305 PMC6489685

[39] Piquer J Joanes V Roldan P Barcia-Salorio JL Masbout G . Long-term results of percutaneous gasserian ganglion lesions. Acta Neurochir Suppl (Wien). (1987) 39:139–41. doi: 10.1007/978-3-7091-8909-2_36, PMID: 3499762

[40] Yuan J Cao S Huang Y Zhang Y Xie P Zhang Y . Altered spontaneous brain activity in patients with idiopathic trigeminal neuralgia: A resting-state functional MRI study. Clin J Pain. (2018) 34:600–9. doi: 10.1097/AJP.0000000000000578, PMID: 29252869 PMC5999362

[41] Liu H Hou H Li F Zheng R Zhang Y Cheng J . Structural and functional brain changes in patients with classic trigeminal neuralgia: A combination of voxel-based morphometry and resting-state functional MRI study. Front Neurosci. (2022) 16. doi: 10.3389/fnins.2022.930765, PMID: 35844235 PMC9277055

[42] Wang Y Zhang X Guan Q Wan L Yi Y Liu CF . Altered regional homogeneity of spontaneous brain activity in idiopathic trigeminal neuralgia. Neuropsychiatr Dis Treat. (2015) 11:2659–66. doi: 10.2147/NDT.S94877, PMID: 26508861 PMC4610767

[43] Liang Y Zhao Q Hu Z Bo K Meyyappan S Neubert JK . Imaging the neural substrate of trigeminal neuralgia pain using deep learning. Front Hum Neurosci. (2023) 17:1144159. doi: 10.3389/fnhum.2023.1144159, PMID: 37275345 PMC10232768

[44] Dou Z Zhang X Yang L Wang W Li N Liu Z . Alternation of regional homogeneity in trigeminal neuralgia after percutaneous radiofrequency thermocoagulation: A resting state fMRI study. Med (Baltimore). (2016) 95:e5193. doi: 10.1097/MD.0000000000005193, PMID: 27759655 PMC5079339

[45] Liu W-C Winslow NK Chao L Nersesyan H Zagardo MT Tracy PT . Neural activity in trigeminal neuralgia patients with sensory and motor stimulations: A pilot functional MRI study. Clin Neurol Neurosurg. (2022) 219:107343. doi: 10.1016/j.clineuro.2022.107343, PMID: 35759909

